# Injuries of the Neck of the Femur

**Published:** 1890-03-15

**Authors:** 


### SURGERY.

### INJURIES OF THE NECK OF THE FEMUR.

Mr. Hugh Thomas, of Liverpool, has some observations on this subject in the January number of the *Provincial Medical Journal.* The author remarks that in the past, while treating fractures of the neck of the femur, we have made theoretical errors similar to those committed when treating fractures of the patella, devoting energies and thought to the question of restoration, ignoring that of repair. The old classification of fractures of the neck of the femur into intra-capsular and extra-capsular has been generally abandoned, being inaccurate in the majority of such accidents. Instead of concentrating attention on the relation of the fracture to the capsule of the joint the impaction or non-impaction of the fragments should be considered, and this for an important clinical reason. Impacted fractures, unless becoming unimpacted, are found to unite by bone, and so to be attended with less serious results than the other variety. The great thing to avoid is any manipulation which can separate the two fragments, and, therefore, examination should be conducted in such a way that this cannot happen. With unimpacted fragments the object aimed at is to place the fragments in such firm contact that a kind of slight impaction is produced. As it is mostly elderly persons who are treated for this lesion it is important that means should be used which least hamper them, and that purposeless restraint be refrained from as much as possible. The American surgeon Senn fixes the limb in all cases with plaster of Paris splint, but to this Mr. Thomas objects. He employs a "hip-splint," similar to that used for ordinary cases of hip-joint disease. The essentials of treatment are to arrest flexion of the hip-joint; to continue the treatment until the symptoms of genuine repair and soundness of the joint are diagnosed. And as with patellar fractures so with these, the younger the patientt he l onger the period of restraint. But the surgeon has first to take into consideration the patient's age. If under, or even at his prime period, details of treatment are those of fractures generally. But at the declining period of life the aim should be to curtail the period of treatment, and the surgeon may find it advisable to ignore any shortening, devoting his energies only to early repair. Under the idea that fracture of the neck of the femur high up must unite by ligament, such injuries have sometimes received no methodical treatment or mechanical assistance. The treatment of fracture of the neck of the femur has never been very satisfactory, and we very much doubt whether any method of mechanical restraint, whether by Senn's plaster of Paris, or by the author's hip joint disease splint, will materially aid the cure in the old and feeble.

